# Inflammation-Related Gene Polymorphisms Associated With Primary Immune Thrombocytopenia

**DOI:** 10.3389/fimmu.2017.00744

**Published:** 2017-06-28

**Authors:** Ju Li, Sai Ma, Linlin Shao, Chunhong Ma, Chengjiang Gao, Xiao-hui Zhang, Ming Hou, Jun Peng

**Affiliations:** ^1^Department of Hematology, Qilu Hospital, Shandong University, Jinan, China; ^2^Department of Immunology, Shandong University School of Medicine, Jinan, China; ^3^Beijing Key Laboratory of Hematopoietic Stem Cell Transplantation, Peking University Institute of Hematology, Peking University People’s Hospital, Beijing, China; ^4^Shandong Provincial Key Laboratory of Immunohematology, Qilu Hospital, Shandong University, Jinan, China

**Keywords:** primary immune thrombocytopenia, inflammation, single-nucleotide polymorphism, susceptibility, treatment

## Abstract

Primary immune thrombocytopenia (ITP) is an acquired autoimmune disease characterized by a reduced platelet count and an increased risk of bleeding. Although immense research has improved our understanding of ITP, the pathogenesis remains unclear. Here, we investigated the involvement of 25 single-nucleotide polymorphisms (SNPs) of the inflammation-related genes, including *CD24, CD226, FCRL3, IL2, IRF5, ITGAM, NLRP3, CARD8, PTPN22, SH2B2, STAT4, TNFAIP3*, and *TRAF1*, in the pathogenesis and treatment response of ITP. We recruited 312 ITP inpatients and 154 healthy participants in this case–control study. Inflammation-related SNP genotyping was performed on the Sequenom MassARRAY iPLEX platform. The expression of TNFAIP3 mRNA was determined by quantitative real-time RT-PCR. All SNPs in healthy controls were consistent with Hardy–Weinberg equilibrium. Statistical analysis revealed that rs10499194 in *TNFAIP3* was significantly associated with a decreased risk of ITP after Bonferroni multiple correction (codominant, CT vs. CC, OR = 0.431, 95% CI = 0.262–0.711, *p* = 0.001; dominant, TT/CT vs. CC, OR = 0.249, 95% CI = 0.141–0.440, *p* = 0.000). Besides, *TNFAIP3* expression was significantly higher in patients with CT and pooled CT/TT genotypes compared with CC genotype (*p* = 0.001; *p* = 0.001). Interestingly, rs10499194 was also associated with corticosteroid-sensitivity (codominant, CT vs. CC, OR = 0.092, 95% CI = 0.021–0.398, *p* = 0.001; dominant, TT/CT vs. CC, OR = 0.086, 95% CI = 0.020–0.369, *p* = 0.001; allelic, T vs. C, OR = 0.088, 95% CI = 0.021–0.372, *p* = 0.001). Furthermore, no significant association was found between inflammation-related SNPs and the severity or refractoriness of ITP after Bonferroni multiple correction. Our findings suggest that rs10499194 may be a protective factor for susceptibility and corticosteroid sensitivity in ITP patients.

## Introduction

Primary immune thrombocytopenia (ITP) is an acquired autoimmune disease characterized by a reduced platelet count and an increased risk of bleeding (1). The pathogenesis of ITP includes enhanced platelet clearance and impaired platelet production, which is related to T cell-mediated effects, B cell-mediated effects, tolerance checkpoint defects, and more (2, 3). Although immense research has improved our understanding of ITP, the etiology is still not completely clear. However, it has been widely accepted that both genetic and environmental factors play a significant role in the pathogenesis of ITP (4).

Autoimmune disease is characterized by self-antigen-induced chronic activation of immune system and eventually leads to tissue inflammation in genetically predisposed individuals (5). ITP is an autoimmune disease and closely related to inflammation (6, 7). Besides, inflammation can trigger ITP. For example, serum uric acid, an inflammatory mediator, played a pathophysiological role in the occurrence of ITP (8). However, ITP can also induce inflammation through platelet dysfunction. Recent research revealed that the acute phase of platelet destruction in severe ITP may trigger inflammation in the lung, which may induce interstitial lung disease (9). Furthermore, platelets mediate inflammation and immune-mediated disorders through multiple mechanisms, such as release of pro-inflammatory mediators, surface inflammation-related molecules, and interaction with leukocytes and endothelial cells (10, 11). Even after being phagocytosed by the mononuclear phagocyte system, platelets modulated survival surface molecules in monocytes (12). In others words, platelets regulated inflammation even after being eliminated from the circulation.

Single-nucleotide polymorphisms (SNPs), the most common pattern of genetic variants, have drawn attention because of their universal distribution and gradually uncovered biological functions (13). Many SNPs of inflammatory cytokine genes, including *IL-17F, IL-10, TNFA, TNFB, TGF-beta 1, IL-6, INF-gamma*, and *IL-1A*, influence patients’ susceptibility to ITP (14–18). Among them, *IL-17F* rs763780 G allele has been identified as a protective factor in ITP, while *TNFB* + 252G/A A allele was a risk factor. There were also some SNPs associated with the severity of ITP. *IL-10* rs1800872 AA genotype presented a significant association with severity of ITP in a cohort of Egyptian population (*p* = 0.008) (14). The *IL-10* gene polymorphism was also identified to be associated with the severity in Japanese population (*p* = 0.01) (19). When it comes to corticosteroid sensitivity of ITP patients, an earlier study reported that stromal cell-derived factor-1 (*SDF-1*) rs2297630 GA and AA genotypes significantly increased the corticosteroid treatment sensitivity compared with GG genotype (*p* = 0.007) (20). Interestingly, a recent study showed that this SDF-1 polymorphism was associated with corticosteroid dependence in pediatric ITP patients (21). Furthermore, researchers identified that ATP-binding cassette gene B1 gene (*ANCB1*) G2677T/A polymorphism was significantly associated with corticosteroid sensitivity (22). Besides, there were several researches on the association between SNPs and the refractoriness of ITP. For example, it was identified that *TNFA*-308G/A A allele was a risk factor for refractory ITP (17). In addition, genetic variants of a cluster of inflammation-related genes, including *CD24, CD226, FCRL3, IL2, IRF5, ITGAM, NLRP3, CARD8, PTPN22, SH2B3, STAT4, TNFAIP3*, and *TRAF1*, are associated with diverse autoimmune diseases. For example, the rs8734 polymorphism in *CD24* was associated with susceptibility to diverse autoimmune diseases including rheumatoid arthritis (RA), autoimmune thyroid disease (AITD), systemic lupus erythematosus (SLE), multiple sclerosis (MS), and inflammatory bowel disease (IBD) (23–29). In addition, four SNP candidates in *FCRL3*, including rs11264799, rs7528684, rs945635, and rs3761959, have been identified as potential risk factors in multiple autoimmune diseases, including RA, AITD, SLE, MS, autoimmune Addison’s disease, and autoimmune pancreatitis (30–36). Finally, rs6822844, located between the *IL2* gene and the nearby *IL21* gene, was associated with RA, IBD, type 1 diabetes, and psoriasis (37–40). Therefore, there are multiple inflammation-related SNPs that are robustly associated with multiple autoimmune diseases. The above studies demonstrated that multiple genetic factors involved in the pathogenesis of ITP and other autoimmune diseases. However, most previous researches only verified the associations between SNPs and ITP susceptibility or ITP treatment. None of them associated the polymorphisms with complete clinical data including susceptibility, severity, corticosensitivity, and refractoriness.

Given that ITP is an inflammatory and autoimmune disease and given the above associations between inflammation-related SNPs and other autoimmune diseases, we hypothesized that these SNPs were also associated with primary ITP. The aim of our study was to investigate the association between inflammation-related gene polymorphisms and the pathogenesis of primary ITP in the Chinese Han population.

## Materials and Methods

### Study Participants

In this case–control study, 312 ITP inpatients were recruited between January 2007 and April 2016 from the Department of Hematology, Qilu Hospital, Shandong University, Jinan, China.

Patients were diagnosed with primary ITP according to the International Working Group guidelines (41). And other autoimmune diseases or underlying immune dysregulation were excluded by medical history, clinical manifestations, physical examinations, radiologic findings and laboratory findings such as HIV and HCV testing, direct antiglobulin test, antiphospholipid antibody testing, antinuclear antibody testing, antithyroid antibody, and thyroid function testing, and testing for other acute and persistent infections. Cases were stratified by severity, corticosteroid sensitivity, and refractoriness based on the standardized definitions (1). Severe ITP is defined by the presence of bleeding symptoms at presentation sufficient to mandate treatment, or by the occurrence of new bleeding symptoms requiring additional therapeutic intervention with a different platelet-enhancing agent or an increased dose to relieve symptoms caused by low platelet count (platelet count may even <10 × 10^9^/L). The corticosteroid regimen is dexamethasone 40 mg p.o. daily for four consecutive days (non-responders received an additional 4-day course of dexamethasone) or prednisone 1.0 mg/kg body weight p.o. daily for four consecutive weeks. Corticosteroid sensitivity is defined as a platelet count ≥30 × 10^9^/L with at least a twofold increase from the baseline count and without bleeding after corticosteroid management. Requirements for additional interventions were considered as corticosteroid resistance. Refractory ITP patients satisfy two criteria. First, they should fail splenectomy. Second, they either manifest severe ITP or have a risk of bleeding that required therapy in the opinion of the attending physician.

For the control group, 205 healthy participants were enrolled. Controls were randomly selected from healthy volunteers with no symptoms of ITP and no history of other autoimmune diseases. All participants were Han Chinese and no genetic associations were found between any participants.

The study was approved by the Medical Ethical Committee of Qilu Hospital, Shandong University. Written informed consent was obtained from each participant in accordance with the Declaration of Helsinki.

### DNA Extraction and Genotyping

Genomic DNA was extracted from peripheral blood mononuclear cells (PBMCs) that had been isolated from whole blood samples (5 mL) from participants by a standard protocol. DNA concentration and purity were accessed at 260/280 absorbance by a NanoDrop spectrophotometer. The DNA extraction was stored at −20°C until genotyping. Inflammation-related SNPs (summarized in Table 1) genotyping was performed on a MassArray system (Sequenom iPLEXassay, BGI Tech., Beijing, China), which is based on a multiplex PCR reaction, a locus-specific single-base extension reaction, and matrix-assisted laser desorption ionization time-of-flight mass spectrometry.

**Table 1:** Selected genes and SNPs.

Genes	SNPs
CD24	rs8734
FCRL3	rs11264799, rs7528684, rs945635, rs3761959
CD226	rs763361
IL2	rs6822844
IRF5	rs2004640, rs2280714, rs10954213
ITGAM	rs1143679
NLRP3	rs35829419, rs4353135, rs10754558
CARD8	rs2043211
PTPN22	rs33996649, rs1310182
SH2B3	rs3184504
STAT4	rs7574865, rs10181656
TNFAIP3	rs6920220, rs10499194, rs2230926, rs5029939
TRAF1	rs10818488

*SNP, single-nucleotide polymorphism*.

### Statistical Analysis

The *p* value of the Hardy–Weinberg equilibrium (HWE) was calculated using the calculator available at the Helmholtz Center Munich website. In addition to allelic frequencies, we analyzed genotypic frequencies under three genetic models, specifically, the codominant, dominant, and recessive models. Associations between the SNPs and ITP susceptibility, severity, corticosteroid sensitivity, and refractoriness were calculated by a chi-squared (χ^2^) test or a Fisher’s exact test. Univariate and multivariate binary logistic regression analyses were used to analyze adjusted *p* values and odds ratios (ORs) with a corresponding 95% confidence interval (95% CI). A two-tailed *p* < 0.05 (or adjusted *p* value by Bonferroni multiple testing) was considered statistically significant. All statistical analyzes were performed using SPSS 22.0 software (SPSS Inc., Chicago, IL, USA).

### RNA Extraction and Real-time RT-PCR of TNFAIP3

Total RNA of PBMCs was isolated by TRIzol reagent (Invitrogen). RNA was converted into cDNA using the PrimeScript RT reagent kit (Perfect Real Time; Takara) according to the manufacturer’s instructions. Multiplex real-time RT-PCR was performed for *TNFAIP3* and the endogenous control β-actin on an ABI PRISM_7500 Sequence Detection System (Applied Biosystems) using SYBR Green (Toyobo) as a double-strand DNA-specific binding dye. The primers for all mRNA assays were intron spanning. The PCR reactions were cycled 40 times after initial denaturation (95°C, 10 min) at 95°C for 15 s and at 60°C for 30 s. The primers for *TNFAIP3* and β-actin are as follows: *TNFAIP3* forward: GTGTATTTTGGGACTCCAGA, *TNFAIP3* reverse: ACTTCTGGCAGTATCCTTCA; β-actin forward: CACCAACTGGGACGACAT, β-actin reverse: GCACAGCCTGGATAGCAAC. We used the comparative threshold cycle (*C*_t_) method for relative quantification of TNFAIP3 mRNA according to relative expression software tool (Michael) (42). The amplification efficiency between the target (*TNFAIP3*) and the reference control (β-actin) were compared to use the delta *C*_t_ (ΔΔ*C*_t_) calculation.

## Results

### Study Population

Demographic and clinical characteristics of controls and ITP patients are summarized in Table 2. All inflammation-related SNPs in the control group were in accordance with HWE. No significant deviations were observed after Bonferroni multiple correction (*p* > 0.002, Table S1 in Supplementary Material).

**Table 2:** Demographic and clinical characteristics.

	Controls	ITP patients
No.	205	312
Age, mean ± SD	45.72 ± 13.41	39.96 ± 13.69
Gender (M/F)	75/130	116/196
ITP severity, *n* (%)		
Severe ITP	NA	189 (60.6)
Non-severe ITP	NA	123 (39.4)
Treatment, *n* (%)		
No use of corticosteroid	NA	50 (16.0)
Corticosteroid-sensitive	NA	156 (50.0)
Corticosteroid-resistant	NA	106 (34.0)
Refractory ITP	NA	21 (6.7)
Non-refractory ITP	NA	291 (93.3)

*M, male; F, female; NA, not applicable; ITP, immune thrombocytopenia*.

### Association between Inflammation-Related SNPs and ITP Susceptibility

Four genetic models were used to analyze the association between the 25 inflammation-related SNPs and ITP. We analyzed the relationship between every single locus and the susceptibility to ITP by a chi-squared (χ^2^) test or a Fisher’s exact test (Table S1 in Supplementary Material). Preliminary screening showed that allelic frequencies of rs8734 in *CD24* and genotypic frequencies of rs11264799 in *FCRL3* under the codominant model were significantly associated with susceptibility to ITP (*p* < 0.05). In addition, both the allelic and genotypic frequencies of rs10499194 in *TNFAIP3* under the codominant and dominant models were significantly associated with the susceptibility to ITP (*p* < 0.05). Among the above SNPs, only rs10499194 was associated with susceptibility of ITP after Bonferroni multiple correction.

Univariate logistic regression analysis revealed that for rs8734 in *CD24*, allele A in place of G was significantly associated with susceptibility to ITP after adjusting for age and gender (*p* = 0.035, Table 3). For rs11264799 in *FCRL3*, the CT rather than CC genotype was significantly associated with susceptibility to ITP under the codominant model (*p* = 0.037, Table 3). For rs10499194 in *TNFAIP3*, CT and CC/CT genotypes were both statistically significant compared to CC (*p* = 0.001 and *p* = 0.000, respectively, Table 3). rs10499194 allelic distribution also showed a statistically significant difference (*p* = 0.003; Table 3). Interestingly, all three inflammation-related polymorphisms demonstrated a protective effect. Among these polymorphisms, only rs10499194 presented a significant association with susceptibility to ITP with univariate logistic regression analysis after Bonferroni multiple correction.

**Table 3:** Association between selected SNPs and ITP risk.

Gene	SNP	Model/allele	Genotype/allele	Controls		ITP patients		OR (95% CI)	Adjusted *p* value
				Count	%	Count	%		
CD24	rs52812045	Allele	G	244	59.5	411	65.9	1.000	
			A	166	40.5	213	34.1	0.754 (0.580–0.981)	**0.035**
FCRL3	rs11264799	Codominant	CC	119	58.0	205	65.7	1.000	
			TT	3	1.5	11	3.5	0.544 (0.678–9.547)	0.166
			CT	83	40.5	96	30.8	0.668 (0.458–0.975)	**0.037**
TNFAIP3	rs10499194	Codominant	CC	160	78.0	277	88.8	1.000	
			TT	1	0.5	2	0.6	1.235 (0.106–14.385)	0.866
			CT	44	21.5	33	10.6	0.431 (0.262–0.711)	**0.001**
		Dominant	CC	160	78.0	292	93.6	1.000	
			TT/CT	45	22.0	20	6.4	0.249 (0.141–0.440)	**0.000**
		Allele	C	364	88.8	587	94.1	1.000	
			T	46	11.2	37	5.9	0.499 (0.315–0.791)	**0.003**

*SNP, single-nucleotide polymorphism; CI, confidence interval; OR, odds ratio; ITP, immune thrombocytopenia*.

*Adjusted p value calculated with univariate logistic regression*.

*Bold highlights statistical significance (*p* < 0.05)*.

Next, we performed a multivariate logistic regression analysis under the codominant model. We found that the heterozygous genotypes of *FCRL3* rs11264799 and *TNFAIP3* rs10499194 significantly decreased the risk of ITP compared with homozygous major alleles (*p* = 0.029 and *p* = 0.001, respectively, Table 4). When we analyzed the combined influence of allelic distribution of both *CD24* rs52812045 and *TNFAIP3* rs10499194, we found a statistical difference in *TNFAIP3* rs10499194 between ITP patients and controls (*p* = 0.006, Table 5).

**Table 4:** Association between selected SNPs and ITP risk under codominant model.

Genes	SNP	Genotype	Controls		ITP patients		OR (95% CI)	Adjusted *p* value
			Count	%	Count	%		
FCRL3	rs11264799	CC	119	58.0	205	65.7	1.000	
		TT	3	1.5	11	3.5	2.703 (0.719–10.161)	0.141
		CT	83	40.5	96	30.8	0.653 (0.446–0.958)	**0.029**
TNFAIP3	rs10499194	CC	160	78.0	277	88.8	1.000	
		TT	1	0.5	2	0.6	1.285 (0.104–15.869)	0.845
		CT	44	21.5	33	10.6	0.415 (0.250–0.689)	**0.001**

*SNP, single-nucleotide polymorphism; CI, confidence interval; OR, odds ratio; ITP, immune thrombocytopenia*.

*Adjusted p value calculated with multivariate logistic regression*.

*Bold highlights statistical significance (*p* < 0.05)*.

**Table 5:** Association between selected SNPs and ITP risk in allelic analysis.

Genes	SNP	Allele	Controls		ITP patients		OR (95% CI)	Adjusted *p* value
			Count	%	Count	%		
CD24	rs52812045	G	244	59.5	411	65.9	1	
		A	166	40.5	213	34.1	0.785 (0.0620–1.023)	0.073
TNFAIP3	rs10499194	C	364	88.8	587	94.1	1.000	
		T	46	11.2	37	5.9	0.522 (0.329–0.829)	**0.006**

*SNP, single-nucleotide polymorphism; CI, confidence interval; OR, odds ratio; ITP, immune thrombocytopenia*.

*Adjusted p value calculated with multivariate logistic regression*.

*Bold highlights statistical significance (*p* < 0.05)*.

### Association between Inflammation-Related SNPs and ITP Severity

We next evaluated the association of the inflammation-related SNPs with ITP disease severity. *CARD8* rs2043211 and *TRAF1* rs10818488 genotypes were statistically different between severe and non-severe ITP patients according to chi-squared analyses (*p* < 0.05, Table S2 in Supplementary Material). However, neither difference persisted after Bonferroni multiple correction. *CARD8* rs2043211 lost the statistical difference after adjusting for age and gender factors by univariate binary logistic regression under dominant model (AA + AT vs. TT: OR = 0.589, 95% CI = 0.343–1.013, *p* = 0.056). After adjusting for age and gender, ITP patients carrying the AG genotype of *TRAF1* rs10818488 showed a 1.713-fold increased risk of developing severe ITP compared with patients carrying major genotype GG under codominant model (OR = 1.713, 95% CI = 1.008–2.911, *p* = 0.047). In summary, only the AG genotype of *TRAF1* rs10818488 increased the risk of severe ITP among patients.

### Association between Inflammation-Related SNPs and Corticosteroid Sensitivity

After studying associations with the pathogenesis of ITP, we explored the association between the inflammation-related polymorphisms and the treatment of ITP. Specifically, we studied corticosteroid sensitivity and refractoriness among patients with different genotypes or alleles. To study corticosteroid sensitivity, we divided the patients who received corticosteroid treatment into two groups, the corticosteroid-sensitive group (*n* = 156) and the corticosteroid-resistant group (*n* = 106).

We found statistically significant associations between inflammation-related polymorphisms and corticosteroid sensitivity. rs945635, rs7528684, and rs3761959 of *FCRL3* and rs2004640 of *IRF5* under the dominant model were significantly associated with corticosteroid-sensitivity (*p* < 0.05, Table S3 in Supplementary Material). rs4353135 of *NLRP3* under both the codominant and recessive models showed significant association with corticosteroid sensitivity (*p* < 0.05, Table S3 in Supplementary Material). Allelic frequencies and genotypic frequencies under the codominant and dominant models of *TNFAIP3* rs10499194 revealed statistically significant differences between the corticosteroid-sensitive and corticosteroid-resistant groups (*p* < 0.05, Table S3 in Supplementary Material). *TNFAIP3* rs10499194 remained significant after Bonferroni multiple correction.

Allelic and genotypic frequencies were analyzed by logistic regression analysis. After univariate logistic regression analysis, compared with major allele homozygotes, minor allele homozygotes, and heterozygotes of *FCRL3* rs945635, rs7528684, and rs3761959 and *IRF* rs2004640 were still significantly associated with corticosteroid-sensitivity under the dominant model after adjusting for age and gender (*p* = 0.029, *p* = 0.029, *p* = 0.029, *p* = 0.024, respectively, Table 6). Genotypic frequencies of *NLRP3* rs4353135 were significantly different under the recessive model between the corticosteroid-sensitive and -resistant groups (*p* = 0.028, Table 6). Genotypic and allelic frequencies of *TNFAIP3* rs10499194 were significantly different under the codominant and dominant models (all *p* = 0.001, Table 6). Importantly, the observed differences in *TNFAIP3* rs10499194 were still statistically significant after Bonferroni multiple correction (*p* < 0.002).

**Table 6:** Association between selected SNPs and corticosteroid-sensitivity of ITP patients.

Gene	SNP	Model/allele	Genotype/allele	Sensitive		Resistant		OR (95% CI)*	*p* Value*	OR (95% CI)^#^	*p* Value^#^
				Count	%	Count	%				
TNFAIP3	rs10499194	Codominant	CC	128	82.1	104	98.1	1			
			TT	2	1.3	0	0.0	–	0.999		
			CT	26	16.7	2	1.9	0.092 (0.021–0.398)	**0.001**		
		Dominant	CC	128	82.1	104	98.1	1.000		1.000	
			TT/CT	28	17.9	2	1.9	0.086 (0.020–0.369)	**0.001**	0.069 (0.016–0.302)	**0.000**
		Allele	C	282	90.4	210	99.1	1.000			
			T	30	9.6	2	0.9	0.088 (0.021–0.372)	**0.001**		
IRF5	rs2004640	Dominant	GG	96	61.5	51	48.1	1.000		1.000	
			TT/GT	60	38.5	55	51.9	1.793 (1.079–2.980)	**0.024**	1.996 (1.162–3.429)	**0.012**
FCRL3	rs3761959	Dominant	CC	65	41.7	30	28.3	1.000		1.000	
			TT/CT	91	58.3	76	71.7	1.807 (1.062–3.076)	**0.029**	2.107 (1.208–3.674)	**0.009**
FCRL3	rs7528684	Dominant	AA	65	41.7	30	28.3	1.000		1.000	
			GG/AG	91	58.3	76	71.7	1.807 (1.062–3.076)	**0.029**	2.107 (1.208–3.674)	**0.009**
FCRL3	rs945635	Dominant	CC	65	41.7	30	28.3	1.000		1.000	
			GG/CG	91	58.3	76	71.7	1.807 (1.062–3.076)	**0.029**	2.107 (1.208–3.674)	**0.009**
NLRP3	rs4353135	Codominant	TT	49	31.4	36	34.0	1.000			
			GG	24	15.4	29	27.4	1.599 (0.798–3.205)	0.186		
			GT	83	53.2	41	38.7	0.684 (0.385–1.212)	0.193		
		Recessive	TT/GT	132	84.6	77	72.6	1.000			
			GG	24	15.4	29	27.4	1.991 (1.077–3.681)	**0.028**		

*SNP, single-nucleotide polymorphism; CI, confidence interval; OR, odds ratio; ITP, immune thrombocytopenia*.

*Calculated by univariate logistic regression.

*^#^Calculated by multivariate logistic regression analysis under dominant model*.

*Bold highlights statistical significance (*p* < 0.05)*.

Multivariate logistic regression showed significant associations between *FCRL3* rs945635, rs7528684, and rs3761959, *IRF* rs2004640, and *TNFAIP3* rs10499194 and corticosteroid sensitivity (*p* = 0.009, *p* = 0.009, *p* = 0.009, *p* = 0.012, and *p* = 0.000, respectively, Table 6). *TNFAIP3* rs10499194 was significantly associated with corticosteroid-sensitivity under both univariate and multivariate logistic regression analyses after Bonferroni multiple correction (*p* < 0.002).

### Association between Inflammation-Related SNPs and ITP Refractoriness

To study refractoriness, we divided the enrolled patients into two subgroups: those refractory to splenectomy treatment and those who responded to medical treatment. We analyzed the association between the inflammation-related SNPs and refractoriness of ITP using chi-squared tests for preliminary screening. The genotypic distribution of *STAT4* rs7574869 was significantly associated with refractoriness under the codominant model (*p* < 0.05, Table S4 in Supplementary Material). No statistical differences were found between other SNPs and refractoriness (*p* > 0.05). However, the association between *STAT4* rs7574869 and refractoriness was not statistically significant after Bonferroni multiple correction. When the GG genotype was used as reference, neither the TT nor GT genotypes were significantly associated with refractoriness after adjusting for the age and gender (*p* = 0.998 and *p* = 0.108, respectively).

### Association of TNFAIP3 rs10499194 Polymorphism and Expression Levels of TNFAIP3

**Figure 1:**
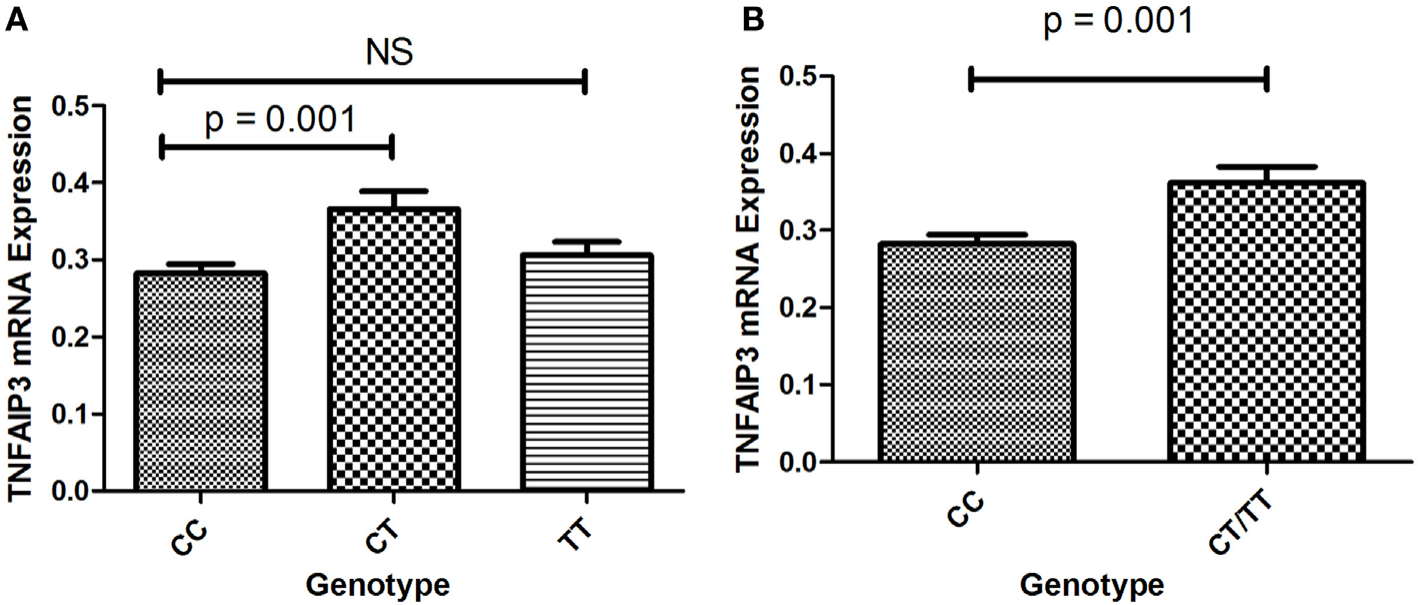
**(A)** Expression of TNFAIP3 mRNA in ITP patients with the CC, CT and TT genotypes. **(B)** Expression of TNFAIP3 mRNA in ITP patients with the CC and pooled CT/TT genotypes.

To determine whether *TNFAIP3* rs10499194 was a protective factor for ITP, we further explored the effects of this polymorphism on *TNFAIP3* expression. The number of ITP patients with TT genotype was small (*n* = 2) in this study, which may lead to an underpowered assessment. The expression of *TNFAIP3* was assessed in 85 ITP patients (60 cases of CC, 2 cases of TT, and 23 cases of CT) by quantitative real-time RT-PCR. As shown in Figure 1A, patients with CT genotype showed higher levels of TNFAIP3 mRNA expression than those with CC genotype (*p* = 0.001). We found that TNFAIP3 expression was significantly higher in samples with pooled CT/TT genotype compared with the CC genotype (*p* = 0.001; Figure 1B). The result showed that CC genotype on *TNFAIP3* rs10499194 was a protective factor for ITP.

## Discussion

Immune thrombocytopenia is an autoimmune and inflammatory disease characterized by immune-mediated platelet destruction. Although ITP has been associated with inflammation, only a few studies have identified the association between inflammation-related SNPs and the pathogenesis of ITP (43). By contrast, inflammation-related gene polymorphisms have been associated with multiple autoimmune diseases.

In this study, we assessed the associations between 25 inflammation-related SNPs and the occurrence and treatment of ITP in the Chinese Han population. Of these SNPs, the frequencies of the homozygous minor allele of *FCRL3* rs11264799 and *TNFAIP3* rs10499194 were significantly decreased in ITP patients. The A allele of *CD24* rs8734 was associated with a decreased risk of ITP. In addition, compared with the CC genotype, individuals carrying pooled TT/CT genotypes of *TNFAIP3* rs10499194 had a decreased risk of ITP under the dominant model. These three SNPs were all protective factors.

Our findings contrast evidence found regarding other autoimmune diseases (26, 44–48). For example, in the Spanish population, no association was found between *FCRL3* rs11264799 and MS (44). Interestingly, however, the C allele of *FCRL3* rs7528684 was a protective factor for MS. This suggests that *FCRL3* polymorphisms may protect individuals from autoimmune diseases, which is somewhat consistent with our findings. In the Israeli population, individuals with *CD24* rs8734 polymorphisms had an increased risk of IBD, ulcerative colitis, and Crohn’s disease (26). In the Chinese population, a significant association was not found between *TNFAIP3* rs10499194 and SLE (45); however, carriers of the CT genotype and combined TT/CT genotype had an increased risk for RA in the Chinese Han population (46). A recent meta-analysis revealed that there was no association between *TNFAIP3* rs10499194 and RA in Europeans, but a significant association in Asians (OR = 1.254, 95% CI = 1.101–1.429, *p* = 6.7 × 10^−4^) (48). The discrepancies between our results and previous research may be attributed to differences in the populations and autoimmune diseases studied.

When it comes to *TNFAIP3* rs10499194, there were several researches identified that it was significantly associated with the susceptibility of autoimmune diseases. Strong associations were observed between *TNFAIP3* rs10499194 and juvenile idiopathic arthritis (OR = 0.74, 95% CI = 0.61–0.91, *p* < 0.004) (49). Prahalad et al demonstrated that *TNFAIP3* rs10499194 had a significant protective effect against childhood onset RA (OR = 0.60, 95% CI = 0.44–0.83, *p* = 0.002) (50). Consistent with our findings, *TNFAIP3* rs2230926 and rs5029939 were significantly different between chronic ITP and control groups (43), suggesting that *TNFAIP3* polymorphisms may affect the susceptibility to ITP. We found that *TNFAIP3* rs10499194 was significantly associated with the susceptibility of ITP even after Bonferroni multiple correction. Therefore, *TNFAIP3* rs10499194 may be an important susceptibility-related SNP for ITP.

To investigate whether *TNFAIP3* rs10499194 was a functional polymorphism, quantitative real-time RT-PCR was performed to evaluate *TNFAIP3* expression. The data showed that individuals with CT genotype on *TNFAIP3* rs10499194 locus showed higher levels of TNFAIP3 mRNA expression compared with the CC genotype, which might play a role in the susceptibility of ITP. It is widely accepted that *TNFAIP3* is a deubiquitinating protein which can deregulate pro-inflammatory signal pathways including NF-κB- and IRF3-dependent gene expression by deubiquitinating specific signaling molecules (51, 52). Some researchers revealed that TNFAIP3 was a central gatekeeper in inflammation and immunity (53). A recent study demonstrated that lack of TNFAIP3 in B cells resulted in overexpression of pro-inflammatory cytokines, which caused inflammation and autoimmunity in aged mice (54). TNFAIP3-deficient B cells displayed enhanced proliferation and development of autoantibodies (55). In our functional analysis, individuals with CT genotype on *TNFAIP3* rs10499194 locus showed higher levels of TNFAIP3 mRNA expression compared with the wild genotype CC, which is in agreement with our genotyping analysis that CT genotype is a protective factor for ITP. However, the mechanism of polymorphism on TNFAIP3 expression awaits further investigations.

Furthermore, we found that the AG genotype of *TRAF1* rs10818488 was significantly associated with increased risk of severe ITP after adjusting for age and gender. These novel findings will be studied further in order to understand the molecular mechanism of these genetic polymorphisms.

In addition to the pathogenesis of ITP, the mechanism of corticosteroid resistance remains poorly understood. In acute lymphoblastic leukemia, the NLRP3-CASP1 inflammasome induced glucocorticoid resistance (56). Overexpression of CASP1 may promote cleavage of the glucocorticoid receptor, which decreased glucocorticoid sensitivity. We identified that *NLRP3* rs4353135 was significantly associated with increased risk of corticosteroid resistance of ITP under the recessive model; however, this observation was not significant after Bonferroni multiple correction. Thus, we propose that the *NLRP3* rs4353135 polymorphism may enhance expression of the NLRP3-CASP1 inflammasome and lead to corticosteroid resistance. Future studies will examine this hypothesis.

Importantly, *TNFAIP3* rs10499194 was associated with corticosteroid-sensitivity after Bonferroni correction. Future work will examine the potential of *TNFAIP3* rs10499194 as a biomarker for corticosteroid sensitivity.

There were some limitations to our study. On the one hand, the participants enrolled in our study were solely from Chinese Han population. Larger trials investigating multi-racial populations are needed to examine whether the associations between the inflammation-related SNPs and ITP exist in the general population. Second, as mentioned earlier, the biological functions and signaling pathways of these SNPs are not yet known and future research with global collaborations are required. Third, there was a potential selection bias in our study. Only inpatients were recruited in our study, as the corticosteroid-sensitivity and refractoriness of outpatients were difficult to follow-up in our hospital. The inpatients have more severe thrombocytopenia and bleeding symptoms. The bleeding symptoms may include gastrointestinal hemorrhage, extensive skin and mucosal hemorrhage, or intracranial hemorrhage. Besides, the 25 susceptibility loci, we tested were selected basing on the pervious reported loci associated with other autoimmune diseases. The candidate gene approach cannot cover all susceptible loci, which may lead to the selection bias.

## Conclusion

Our investigation of SNPs and ITP provides interesting results. We found that inflammation-related SNPs, especially *TNFAIP3* rs10499194, may be genetic risk factors associated with the development and treatment of ITP. Our findings may lead to clinicians screening for these SNPs in order to predict the prognosis and guide the treatment of ITP.

## Statement of Prior Presentation

Part of this study was presented as a poster with a title of “Inflammation-related Gene Polymorphisms Associated with Susceptibility to Primary Immune Thrombocytopenia” (Abstract Code: 3737) and won ASH Abstract Achievement Award at the 58th ASH Annual Meeting and Exposition in San Diego, CA, USA, December 3–6, 2016.

## Ethics Statement

This study was carried out in accordance with the recommendations of Medical Ethical Committee of Qilu Hospital, Shandong University with written informed consent from all subjects. All subjects gave written informed consent in accordance with the Declaration of Helsinki. The protocol was approved by the Medical Ethical Committee of Qilu Hospital, Shandong University.

## Author Contributions

JP, MH, and X-hZ designed research, analyzed data, and wrote the paper; JL performed research, analyzed data, and wrote the paper; SM, LS, CM, and CG performed research and analyzed data.

## Conflict of Interest Statement

The authors declare that the research was conducted in the absence of any commercial or financial relationships that could be construed as a potential conflict of interest.
