# Supplementary material for: Inflammation-Related Gene Polymorphisms Associated With Primary Immune Thrombocytopenia
Source: Front Immunol. 2017 Jun 28;8:744. doi: 10.3389/fimmu.2017.00744 (PMC5487479; doi:10.3389/fimmu.2017.00744)
Supplement: Supplementary file 1 [file Table_1.DOC]

**Supplementary Table S1.** Hardy-Weinberg equilibrium test and association between inflammation-related SNPs and ITP susceptibility.

| Genes | SNPs | Genotype | Allele | Controls |  | Cases |  | HWE -p | Model / allele | Uncorrected p value |
| --- | --- | --- | --- | --- | --- | --- | --- | --- | --- | --- |
|  |  |  |  | Count | % | Count | % |  |  |  |
| CD24 | rs52812045 | GG |  | 72 | 35.1 | 132 | 42.3 | 0.861 | Codominant | 0.098 |
|  |  | AA |  | 33 | 16.1 | 33 | 10.6 |  | Dominant | 0.102 |
|  |  | AG |  | 100 | 48.8 | 147 | 47.1 |  | Recessive | 0.066 |
|  |  |  | G | 244 | 59.5 | 411 | 65.9 |  | Allele | **0.038** |
|  |  |  | A | 166 | 40.5 | 213 | 34.1 |  |  |  |
| CD226 | rs763361 | CC |  | 89 | 43.4 | 149 | 47.8 | 0.018 | Codominant | 0.195 |
|  |  | TT |  | 13 | 6.3 | 29 | 9.3 |  | Dominant | 0.333 |
|  |  | CT |  | 103 | 50.2 | 134 | 42.9 |  | Recessive | 0.229 |
|  |  |  | C | 281 | 68.5 | 432 | 69.2 |  | Allele | 0.813 |
|  |  |  | T | 129 | 31.5 | 192 | 30.8 |  |  |  |
| FCRL3 | rs945635 | CC |  | 58 | 28.3 | 112 | 35.9 | 0.012 | Codominant | 0.076 |
|  |  | GG |  | 29 | 14.1 | 52 | 16.7 |  | Dominant | 0.072 |
|  |  | CG |  | 118 | 57.6 | 148 | 47.4 |  | Recessive | 0.441 |
|  |  |  | C | 234 | 57.1 | 372 | 59.6 |  | Allele | 0.417 |
|  |  |  | G | 176 | 42.9 | 252 | 40.4 |  |  |  |
|  | rs7528684 | GG |  | 29 | 14.1 | 52 | 16.7 | 0.012 | Codominant | 0.076 |
|  |  | AA |  | 58 | 28.3 | 112 | 35.9 |  | Dominant | 0.072 |
|  |  | AG |  | 118 | 57.6 | 148 | 47.4 |  | Recessive | 0.441 |
|  |  |  | G | 176 | 42.9 | 252 | 40.4 |  | Allele | 0.417 |
|  |  |  | A | 234 | 57.1 | 372 | 59.6 |  |  |  |
|  | rs3761959 | CC |  | 57 | 27.8 | 112 | 35.9 | 0.009 | Codominant | 0.067 |
|  |  | TT |  | 29 | 14.1 | 51 | 16.3 |  | Dominant | 0.055 |
|  |  | CT |  | 119 | 58.0 | 149 | 47.8 |  | Recessive | 0.499 |
|  |  |  | C | 233 | 56.8 | 373 | 59.8 |  | Allele | 0.347 |
|  |  |  | T | 177 | 43.2 | 251 | 40.2 |  |  |  |
|  | rs11264799 | CC |  | 119 | 58.0 | 205 | 65.7 | 0.006 | Codominant | **0.039** |
|  |  | TT |  | 3 | 1.5 | 11 | 3.5 |  | Dominant | 0.078 |
|  |  | CT |  | 83 | 40.5 | 96 | 30.8 |  | Recessive | 0.158 |
|  |  |  | C | 321 | 78.3 | 506 | 81.1 |  | Allele | 0.272 |
|  |  |  | T | 89 | 21.7 | 118 | 18.9 |  |  |  |
| IL2 | rs6822844 | GG |  | 204 | 99.5 | 312 | 100.0 | 0.972 | Codominant | 0.397 |
|  |  | GT |  | 1 | 0.5 | 0 | 0.0 |  | Dominant | 0.397 |
|  |  |  | G | 409 | 99.8 | 624 | 100.0 |  | Recessive | _ |
|  |  |  | T | 1 | 0.2 | 0 | 0.0 |  | Allele | 0.397 |
| IRF5 | rs2280714 | CC |  | 30 | 14.6 | 59 | 18.9 | 0.048 | Codominant | 0.118 |
|  |  | TT |  | 61 | 29.8 | 108 | 34.6 |  | Dominant | 0.249 |
|  |  | CT |  | 114 | 55.6 | 145 | 46.5 |  | Recessive | 0.208 |
|  |  |  | C | 174 | 42.4 | 263 | 42.1 |  | Allele | 0.926 |
|  |  |  | T | 236 | 57.6 | 361 | 57.9 |  |  |  |
|  | rs2004640 | GG |  | 118 | 57.6 | 174 | 55.8 | 0.784 | Codominant | 0.512 |
|  |  | TT |  | 11 | 5.4 | 25 | 8.0 |  | Dominant | 0.688 |
|  |  | GT |  | 76 | 37.1 | 113 | 36.2 |  | Recessive | 0.247 |
|  |  |  | G | 312 | 76.1 | 461 | 73.9 |  | Allele | 0.422 |
|  |  |  | T | 98 | 23.9 | 163 | 26.1 |  |  |  |
|  | rs10954213 | GG |  | 43 | 21.0 | 76 | 24.4 | 0.179 | Codominant | 0.191 |
|  |  | AA |  | 50 | 24.4 | 91 | 29.2 |  | Dominant | 0.233 |
|  |  | AG |  | 112 | 54.6 | 145 | 46.5 |  | Recessive | 0.371 |
|  |  |  | A | 212 | 51.7 | 327 | 52.4 |  | Allele | 0.826 |
|  |  |  | G | 198 | 48.3 | 297 | 47.6 |  |  |  |
| ITGAM | rs1143679 | GG |  | 204 | 99.5 | 308 | 98.7 | 0.972 | Codominant | 0.653 |
|  |  | AG |  | 1 | 0.5 | 4 | 1.3 |  | Dominant | 0.658 |
|  |  |  | A | 1 | 0.2 | 4 | 0.6 |  | Recessive | _ |
|  |  |  | G | 409 | 99.8 | 620 | 99.4 |  | Allele | 0.654 |
| NLRP3 | rs4353135 | GG |  | 40 | 19.5 | 71 | 22.8 | 0.89 | Codominant | 0.670 |
|  |  | TT |  | 65 | 31.7 | 97 | 31.1 |  | Dominant | 0.882 |
|  |  | GT |  | 100 | 48.8 | 144 | 46.2 |  | Recessive | 0.380 |
|  |  |  | G | 180 | 43.9 | 286 | 45.8 |  | Allele | 0.542 |
|  |  |  | T | 230 | 56.1 | 338 | 54.2 |  |  |  |
|  | rs35829419 | CC |  | 205 | 100.0 | 312 | 100.0 | _ | Codominant | _ |
|  |  |  | C | 205 | 100.0 | 312 | 100.0 |  | Dominant | _ |
|  |  |  |  |  |  |  |  |  | Recessive | _ |
|  |  |  |  |  |  |  |  |  | Allele | _ |
|  | rs10754558 | CC |  | 76 | 37.1 | 98 | 31.4 | 0.136 | Codominant | 0.348 |
|  |  | GG |  | 40 | 19.5 | 60 | 19.2 |  | Dominant | 0.183 |
|  |  | CG |  | 89 | 43.4 | 154 | 49.4 |  | Recessive | 0.937 |
|  |  |  | C | 241 | 58.8 | 350 | 56.1 |  | Allele | 0.392 |
|  |  |  | G | 169 | 41.2 | 274 | 43.9 |  |  |  |
| CARD8 | rs2043211 | AA |  | 63 | 30.7 | 77 | 24.7 | 0.205 | Codominant | 0.313 |
|  |  | TT |  | 49 | 23.9 | 83 | 26.6 |  | Dominant | 0.491 |
|  |  | AT |  | 93 | 45.4 | 152 | 48.7 |  | Recessive | 0.130 |
|  |  |  | A | 219 | 53.4 | 306 | 49.0 |  | Allele | 0.169 |
|  |  |  | T | 191 | 46.6 | 318 | 51.0 |  |  |  |
| PTPN22 | rs33996649 | CC |  | 205 | 100.0 | 312 | 100.0 | _ | Codominant | _ |
|  |  |  | C | 205 | 100.0 | 312 | 100.0 |  | Dominant | _ |
|  |  |  |  |  |  |  |  |  | Recessive | _ |
|  |  |  |  |  |  |  |  |  | Allele | _ |
|  | rs1310182 | GG |  | 4 | 2.0 | 7 | 2.2 | 0.535 | Codominant | 0.936 |
|  |  | AA |  | 159 | 77.6 | 238 | 76.3 |  | Dominant | 0.736 |
|  |  | AG |  | 42 | 20.5 | 67 | 21.5 |  | Recessive | 1.000 |
|  |  |  | G | 50 | 12.2 | 81 | 13.0 |  | Allele | 0.710 |
|  |  |  | A | 360 | 87.8 | 543 | 87.0 |  |  |  |
| SH2B3 | rs3184504 | CC |  | 204 | 99.5 | 310 | 99.4 | 0.972 | Codominant | 1.000 |
|  |  | CT |  | 1 | 0.5 | 2 | 0.6 |  | Dominant | 1.000 |
|  |  |  | C | 409 | 99.8 | 622 | 99.7 |  | Recessive | _ |
|  |  |  | T | 1 | 0.2 | 2 | 0.3 |  | Allele | 1.000 |
| STAT4 | rs10181656 | CC |  | 89 | 43.4 | 125 | 40.1 | 0.553 | Codominant | 0.729 |
|  |  | GG |  | 21 | 10.2 | 36 | 11.5 |  | Dominant | 0.449 |
|  |  | CG |  | 95 | 46.3 | 151 | 48.4 |  | Recessive | 0.646 |
|  |  |  | C | 273 | 66.6 | 401 | 64.3 |  | Allele | 0.443 |
|  |  |  | G | 137 | 33.4 | 223 | 35.7 |  |  |  |
|  | rs7574869 | GG |  | 89 | 43.4 | 127 | 40.7 | 0.553 | Codominant | 0.795 |
|  |  | TT |  | 21 | 10.2 | 36 | 11.5 |  | Dominant | 0.541 |
|  |  | GT |  | 95 | 46.3 | 149 | 47.8 |  | Recessive | 0.646 |
|  |  |  | G | 273 | 66.6 | 403 | 64.6 |  | Allele | 0.508 |
|  |  |  | T | 137 | 33.4 | 221 | 35.4 |  |  |  |
| TNFAIP3 | rs10499194 | CC |  | 160 | 78.0 | 277 | 88.8 | 0.268 | Codominant | **0.001** |
|  |  | TT |  | 1 | 0.5 | 2 | 0.6 |  | Dominant | **0.000** |
|  |  | CT |  | 44 | 21.5 | 33 | 10.6 |  | Recessive | 0.520 |
|  |  |  | C | 364 | 88.8 | 587 | 94.1 |  | Allele | **0.002** |
|  |  |  | T | 46 | 11.2 | 37 | 5.9 |  |  |  |
|  | rs2230926 | TT |  | 183 | 89.3 | 277 | 88.8 | 0.417 | Codominant | 0.863 |
|  |  | GT |  | 22 | 10.7 | 35 | 11.2 |  | Dominant | 0.863 |
|  |  |  | G | 22 | 5.4 | 35 | 5.6 |  | Recessive | _ |
|  |  |  | T | 388 | 94.6 | 589 | 94.4 |  | Allele | 0.867 |
|  | rs5029939 | CC |  | 179 | 87.3 | 272 | 87.2 | 0.332 | Codominant | 0.963 |
|  |  | CG |  | 26 | 12.7 | 40 | 12.8 |  | Dominant | 0.963 |
|  |  |  | C | 384 | 93.7 | 584 | 93.6 |  | Recessive | _ |
|  |  |  | G | 26 | 6.3 | 40 | 6.4 |  | Allele | 0.965 |
|  | rs6920220 | GG |  | 199 | 97.1 | 309 | 99.0 | 0.832 | Codominant | 0.184 |
|  |  | AG |  | 6 | 2.9 | 3 | 1.0 |  | Dominant | 0.184 |
|  |  |  | G | 404 | 98.5 | 621 | 99.5 |  | Recessive | _ |
|  |  |  | A | 6 | 1.5 | 3 | 0.5 |  | Allele | 0.167 |
| TRAF1 | rs10818488 | GG |  | 51 | 24.9 | 101 | 32.4 | 0.357 | Codominant | 0.145 |
|  |  | AA |  | 45 | 22.0 | 69 | 22.1 |  | Dominant | 0.067 |
|  |  | AG |  | 109 | 53.2 | 142 | 45.5 |  | Recessive | 0.965 |
|  |  |  | A | 199 | 48.5 | 280 | 44.9 |  | Allele | 0.248 |
|  |  |  | G | 211 | 51.5 | 344 | 55.1 |  |  |  |

SNP, single nucleotide polymorphism; Uncorrected p value calculated with chi-squared test; HWE-p, p value of Hardy–Weinberg equilibrium; **Bold** highlights statistical significance (p < 0.05).
