# Supplementary material for: Inflammation-Related Gene Polymorphisms Associated With Primary Immune Thrombocytopenia
Source: Front Immunol. 2017 Jun 28;8:744. doi: 10.3389/fimmu.2017.00744 (PMC5487479; doi:10.3389/fimmu.2017.00744)
Supplement: Supplementary file 2 [file Table_2.DOC]

**Supplementary Table S2**. Association between inflammation-related SNPs and severity of ITP.

| Gene | SNP | Genotype | Allele | Non-severe ITP patients | | Severe ITP patients | | Model / allele | Uncorrected p value |
| --- | --- | --- | --- | --- | --- | --- | --- | --- | --- |
|  |  |  |  | Count | % | Count | % |  |  |
| CD24 | rs52812045 | GG |  | 50 | 40.7 | 82 | 43.4 | Codominant | 0.323 |
|  |  | AA |  | 17 | 13.8 | 16 | 8.5 | Dominant | 0.633 |
|  |  | AG |  | 56 | 45.5 | 91 | 48.1 | Recessive | 0.133 |
|  |  |  | G | 156 | 63.4 | 255 | 67.5 | Allele | 0.298 |
|  |  |  | A | 90 | 36.6 | 123 | 32.5 |  |  |
| CD226 | rs763361 | CC |  | 52 | 42.3 | 97 | 51.3 | Codominant | 0.180 |
|  |  | TT |  | 15 | 12.2 | 14 | 7.4 | Dominant | 0.118 |
|  |  | CT |  | 56 | 45.5 | 78 | 41.3 | Recessive | 0.155 |
|  |  |  | C | 160 | 65.0 | 272 | 72.0 | Allele | 0.067 |
|  |  |  | T | 86 | 35.0 | 106 | 28.0 |  |  |
| FCRL3 | rs945635 | CC |  | 43 | 35.0 | 69 | 36.5 | Codominant | 0.739 |
|  |  | GG |  | 23 | 18.7 | 29 | 15.3 | Dominant | 0.781 |
|  |  | CG |  | 57 | 46.3 | 91 | 48.1 | Recessive | 0.437 |
|  |  |  | C | 143 | 58.1 | 229 | 60.6 | Allele | 0.542 |
|  |  |  | G | 103 | 41.9 | 149 | 39.4 |  |  |
|  | rs7528684 | GG |  | 23 | 18.7 | 29 | 15.3 | Codominant | 0.739 |
|  |  | AA |  | 43 | 35.0 | 69 | 36.5 | Dominant | 0.781 |
|  |  | AG |  | 57 | 46.3 | 91 | 48.1 | Recessive | 0.437 |
|  |  |  | G | 103 | 41.9 | 149 | 39.4 | Allele | 0.542 |
|  |  |  | A | 143 | 58.1 | 229 | 60.6 |  |  |
|  | rs3761959 | CC |  | 43 | 35.0 | 69 | 36.5 | Codominant | 0.663 |
|  |  | TT |  | 23 | 18.7 | 28 | 14.8 | Dominant | 0.781 |
|  |  | CT |  | 57 | 46.3 | 92 | 48.7 | Recessive | 0.365 |
|  |  |  | C | 143 | 58.1 | 230 | 60.8 | Allele | 0.499 |
|  |  |  | T | 103 | 41.9 | 148 | 39.2 |  |  |
|  | rs11264799 | CC |  | 79 | 64.2 | 126 | 66.7 | Codominant | 0.571 |
|  |  | TT |  | 6 | 4.9 | 5 | 2.6 | Dominant | 0.657 |
|  |  | CT |  | 38 | 30.9 | 58 | 30.7 | Recessive | 0.465 |
|  |  |  | C | 196 | 79.7 | 310 | 82.0 | Allele | 0.467 |
|  |  |  | T | 50 | 20.3 | 68 | 18.0 |  |  |
| IL2 | rs6822844 | GG |  | 123 | 100.0 | 189 | 100.0 | Codominant | _ |
|  |  |  | G | 246 | 100.0 | 378 | 100.0 | Dominant | _ |
|  |  |  |  |  |  |  |  | Recessive | _ |
|  |  |  |  |  |  |  |  | Allele | _ |
| IRF5 | rs2280714 | CC |  | 25 | 20.3 | 34 | 18.0 | Codominant | 0.277 |
|  |  | TT |  | 36 | 29.3 | 72 | 38.1 | Dominant | 0.109 |
|  |  | CT |  | 62 | 50.4 | 83 | 43.9 | Recessive | 0.607 |
|  |  |  | C | 112 | 45.5 | 151 | 39.9 | Allele | 0.168 |
|  |  |  | T | 134 | 54.5 | 227 | 60.1 |  |  |
|  | rs2004640 | GG |  | 73 | 59.3 | 101 | 53.4 | Codominant | 0.383 |
|  |  | TT |  | 7 | 5.7 | 18 | 9.5 | Dominant | 0.304 |
|  |  | GT |  | 43 | 35.0 | 70 | 37.0 | Recessive | 0.223 |
|  |  |  | G | 189 | 76.8 | 272 | 72.0 | Allele | 0.176 |
|  |  |  | T | 57 | 23.2 | 106 | 28.0 |  |  |
|  | rs10954213 | GG |  | 30 | 24.4 | 46 | 24.3 | Codominant | 0.413 |
|  |  | AA |  | 31 | 25.2 | 60 | 31.7 | Dominant | 0.214 |
|  |  | AG |  | 62 | 50.4 | 83 | 43.9 | Recessive | 0.992 |
|  |  |  | A | 124 | 50.4 | 203 | 53.7 | Allele | 0.420 |
|  |  |  | G | 122 | 49.6 | 175 | 46.3 |  |  |
| ITGAM | rs1143679 | GG |  | 121 | 98.4 | 187 | 98.9 | Codominant | 1.000 |
|  |  | AG |  | 2 | 1.6 | 2 | 1.1 | Dominant | 1.000 |
|  |  |  | G | 244 | 99.2 | 376 | 99.5 | Recessive | _ |
|  |  |  | A | 2 | 0.8 | 2 | 0.5 | Allele | 1.000 |
| NLRP3 | rs4353135 | GG |  | 25 | 20.3 | 46 | 24.3 | Codominant | 0.573 |
|  |  | TT |  | 37 | 30.1 | 60 | 31.7 | Dominant | 0.756 |
|  |  | GT |  | 61 | 49.6 | 83 | 43.9 | Recessive | 0.409 |
|  |  |  | G | 111 | 45.1 | 175 | 46.3 | Allele | 0.774 |
|  |  |  | T | 135 | 54.9 | 203 | 53.7 |  |  |
|  | rs35829419 | CC |  | 123 | 100.0 | 189 | 100.0 | Codominant | _ |
|  |  |  | C | 246 | 100.0 | 378 | 100.0 | Dominant | _ |
|  |  |  |  |  |  |  |  | Recessive | _ |
|  |  |  |  |  |  |  |  | Allele | _ |
|  | rs10754558 | CC |  | 41 | 33.3 | 57 | 30.2 | Codominant | 0.544 |
|  |  | GG |  | 20 | 16.3 | 40 | 21.2 | Dominant | 0.555 |
|  |  | CG |  | 62 | 50.4 | 92 | 48.7 | Recessive | 0.283 |
|  |  |  | C | 144 | 58.5 | 206 | 54.5 | Allele | 0.320 |
|  |  |  | G | 102 | 41.5 | 172 | 45.5 |  |  |
| CARD8 | rs2043211 | AA |  | 31 | 25.2 | 46 | 24.3 | Codominant | 0.110 |
|  |  | TT |  | 25 | 20.3 | 58 | 30.7 | Dominant | **0.043** |
|  |  | AT |  | 67 | 54.5 | 85 | 45.0 | Recessive | 0.863 |
|  |  |  | A | 129 | 52.4 | 177 | 46.8 | Allele | 0.170 |
|  |  |  | T | 117 | 47.6 | 201 | 53.2 |  |  |
| PTPN22 | rs33996649 | CC |  | 123 | 100.0 | 189 | 100.0 | Codominant | _ |
|  |  |  | C | 246 | 100.0 | 378 | 100.0 | Dominant | _ |
|  |  |  |  |  |  |  |  | Recessive | _ |
|  |  |  |  |  |  |  |  | Allele | _ |
|  | rs1310182 | GG |  | 3 | 2.4 | 4 | 2.1 | Codominant | 0.935 |
|  |  | AA |  | 95 | 77.2 | 143 | 75.7 | Dominant | 0.749 |
|  |  | AG |  | 25 | 20.3 | 42 | 22.2 | Recessive | 1.000 |
|  |  |  | G | 31 | 12.6 | 50 | 13.2 | Allele | 0.820 |
|  |  |  | A | 215 | 87.4 | 328 | 86.8 |  |  |
| SH2B3 | rs3184504 | CC |  | 123 | 100.0 | 187 | 98.9 | Codominant | 0.521 |
|  |  | CT |  | 0 | 0.0 | 2 | 1.1 | Dominant | 0.521 |
|  |  |  | C | 246 | 100.0 | 376 | 99.5 | Recessive | _ |
|  |  |  | T | 0 | 0.0 | 2 | 0.5 | Allele | 0.522 |
| STAT4 | rs10181656 | CC |  | 51 | 41.5 | 74 | 39.2 | Codominant | 0.662 |
|  |  | GG |  | 16 | 13.0 | 20 | 10.6 | Dominant | 0.684 |
|  |  | CG |  | 56 | 45.5 | 95 | 50.3 | Recessive | 0.512 |
|  |  |  | C | 158 | 64.2 | 243 | 64.3 | Allele | 0.988 |
|  |  |  | G | 88 | 35.8 | 135 | 35.7 |  |  |
|  | rs7574869 | GG |  | 53 | 43.1 | 74 | 39.2 | Codominant | 0.523 |
|  |  | TT |  | 16 | 13.0 | 20 | 10.6 | Dominant | 0.489 |
|  |  | GT |  | 54 | 43.9 | 95 | 50.3 | Recessive | 0.512 |
|  |  |  | G | 160 | 65.0 | 243 | 64.3 | Allele | 0.847 |
|  |  |  | T | 86 | 35.0 | 135 | 35.7 |  |  |
| TNFAIP3 | rs10499194 | CC |  | 107 | 87.0 | 170 | 89.9 | Codominant | 0.685 |
|  |  | TT |  | 1 | 0.8 | 1 | 0.5 | Dominant | 0.419 |
|  |  | CT |  | 15 | 12.2 | 18 | 9.5 | Recessive | 1.000 |
|  |  |  | C | 229 | 93.1 | 358 | 94.7 | Allele | 0.403 |
|  |  |  | T | 17 | 6.9 | 20 | 5.3 |  |  |
|  | rs2230926 | TT |  | 106 | 86.2 | 171 | 90.5 | Codominant | 0.240 |
|  |  | GT |  | 17 | 13.8 | 18 | 9.5 | Dominant | 0.240 |
|  |  |  | G | 17 | 6.9 | 18 | 4.8 | Recessive | _ |
|  |  |  | T | 229 | 93.1 | 360 | 95.2 | Allele | 0.254 |
|  | rs5029939 | CC |  | 106 | 86.2 | 166 | 87.8 | Codominant | 0.670 |
|  |  | CG |  | 17 | 13.8 | 23 | 12.2 | Dominant | 0.670 |
|  |  |  | C | 229 | 93.1 | 355 | 93.9 | Recessive | _ |
|  |  |  | G | 17 | 6.9 | 23 | 6.1 | Allele | 0.681 |
|  | rs6920220 | GG |  | 122 | 99.2 | 187 | 98.9 | Codominant | 1.000 |
|  |  | AG |  | 1 | 0.8 | 2 | 1.1 | Dominant | 1.000 |
|  |  |  | G | 245 | 99.6 | 376 | 99.5 | Recessive | _ |
|  |  |  | A | 1 | 0.4 | 2 | 0.5 | Allele | 1.000 |
| TRAF1 | rs10818488 | GG |  | 45 | 36.6 | 56 | 29.6 | Codominant | **0.035** |
|  |  | AA |  | 33 | 26.8 | 36 | 19.0 | Dominant | 0.199 |
|  |  | AG |  | 45 | 36.6 | 97 | 51.3 | Recessive | 0.106 |
|  |  |  | A | 111 | 45.1 | 169 | 44.7 | Allele | 0.919 |
|  |  |  | G | 135 | 54.9 | 209 | 55.3 |  |  |

SNP, single nucleotide polymorphism; Uncorrected p value calculated with chi-squared test; **Bold** highlights statistical significance (p < 0.05).
