# Supplementary material for: Inflammation-Related Gene Polymorphisms Associated With Primary Immune Thrombocytopenia
Source: Front Immunol. 2017 Jun 28;8:744. doi: 10.3389/fimmu.2017.00744 (PMC5487479; doi:10.3389/fimmu.2017.00744)
Supplement: Supplementary file 3 [file Table_3.DOC]

**Supplementary Table S3**. Association between inflammation-related SNPs and corticosteroid-sensitivity of ITP.

| Gene | SNP | Genotype | Allele | Corticosteroid-sensitive | | Corticosteroid-resistant | | Model / allele | Uncorrected p value |
| --- | --- | --- | --- | --- | --- | --- | --- | --- | --- |
|  |  |  |  | Count | % | Count | % |  |  |
| CD24 | rs52812045 | AA |  | 15 | 9.6 | 10 | 9.4 | Codominant | 0.598 |
|  |  | GG |  | 64 | 41.0 | 50 | 47.2 | Dominant | 0.325 |
|  |  | AG |  | 77 | 49.4 | 46 | 43.4 | Recessive | 0.961 |
|  |  |  | G | 205 | 65.7 | 146 | 68.9 | Allele | 0.450 |
|  |  |  | A | 107 | 34.3 | 66 | 31.1 |  |  |
| CD226 | rs763361 | CC |  | 77 | 49.4 | 50 | 47.2 | Codominant | 0.747 |
|  |  | TT |  | 12 | 7.7 | 11 | 10.4 | Dominant | 0.728 |
|  |  | CT |  | 67 | 42.9 | 45 | 42.5 | Recessive | 0.451 |
|  |  |  | C | 221 | 70.8 | 145 | 68.4 | Allele | 0.551 |
|  |  |  | T | 91 | 29.2 | 67 | 31.6 |  |  |
| FCRL3 | rs945635 | CC |  | 65 | 41.7 | 30 | 28.3 | Codominant | 0.087 |
|  |  | GG |  | 23 | 14.7 | 19 | 17.9 | Dominant | **0.027** |
|  |  | CG |  | 68 | 43.6 | 57 | 53.8 | Recessive | 0.491 |
|  |  |  | C | 198 | 63.5 | 117 | 55.2 | Allele | 0.058 |
|  |  |  | G | 114 | 36.5 | 95 | 44.8 |  |  |
|  | rs7528684 | GG |  | 23 | 14.7 | 19 | 17.9 | Codominant | 0.087 |
|  |  | AA |  | 65 | 41.7 | 30 | 28.3 | Dominant | **0.027** |
|  |  | AG |  | 68 | 43.6 | 57 | 53.8 | Recessive | 0.491 |
|  |  |  | G | 114 | 36.5 | 95 | 44.8 | Allele | 0.058 |
|  |  |  | A | 198 | 63.5 | 117 | 55.2 |  |  |
|  | rs3761959 | CC |  | 65 | 41.7 | 30 | 28.3 | Codominant | 0.087 |
|  |  | TT |  | 23 | 14.7 | 19 | 17.9 | Dominant | **0.027** |
|  |  | CT |  | 68 | 43.6 | 57 | 53.8 | Recessive | 0.491 |
|  |  |  | C | 198 | 63.5 | 117 | 55.2 | Allele | 0.058 |
|  |  |  | T | 114 | 36.5 | 95 | 44.8 |  |  |
|  | rs11264799 | CC |  | 107 | 68.6 | 64 | 60.4 | Codominant | 0.378 |
|  |  | TT |  | 5 | 3.2 | 5 | 4.7 | Dominant | 0.171 |
|  |  | CT |  | 44 | 28.2 | 37 | 34.9 | Recessive | 0.765 |
|  |  |  | C | 258 | 82.7 | 165 | 77.8 | Allele | 0.166 |
|  |  |  | T | 54 | 17.3 | 47 | 22.2 |  |  |
| IL2 | rs6822844 | GG |  | 156 | 100.0 | 106 | 100.0 | Codominant | _ |
|  |  |  | G | 312 | 100.0 | 212 | 100.0 | Dominant | _ |
|  |  |  |  |  |  |  |  | Recessive | _ |
|  |  |  |  |  |  |  |  | Allele | _ |
| IRF5 | rs2280714 | CC |  | 31 | 19.9 | 16 | 15.1 | Codominant | 0.599 |
|  |  | TT |  | 56 | 35.9 | 39 | 36.8 | Dominant | 0.882 |
|  |  | CT |  | 69 | 44.2 | 51 | 48.1 | Recessive | 0.323 |
|  |  |  | C | 131 | 42.0 | 83 | 39.2 | Allele | 0.517 |
|  |  |  | T | 181 | 58.0 | 129 | 60.8 |  |  |
|  | rs2004640 | TT |  | 11 | 7.1 | 8 | 7.5 | Codominant | 0.085 |
|  |  | GG |  | 96 | 61.5 | 51 | 48.1 | Dominant | **0.032** |
|  |  | GT |  | 49 | 31.4 | 47 | 44.3 | Recessive | 0.879 |
|  |  |  | G | 241 | 77.2 | 149 | 70.3 | Allele | 0.073 |
|  |  |  | T | 71 | 22.8 | 63 | 29.7 |  |  |
|  | rs10954213 | GG |  | 35 | 22.4 | 29 | 27.4 | Codominant | 0.659 |
|  |  | AA |  | 51 | 32.7 | 32 | 30.2 | Dominant | 0.669 |
|  |  | AG |  | 70 | 44.9 | 45 | 42.5 | Recessive | 0.363 |
|  |  |  | G | 140 | 44.9 | 103 | 48.6 | Allele | 0.403 |
|  |  |  | A | 172 | 55.1 | 109 | 51.4 |  |  |
| ITGAM | rs1143679 | AG |  | 0 | 0.0 | 3 | 2.8 | Codominant | 0.128 |
|  |  | GG |  | 156 | 100.0 | 103 | 97.2 | Dominant | 0.128 |
|  |  |  | G | 312 | 100.0 | 209 | 98.6 | Recessive | _ |
|  |  |  | A | 0 | 0.0 | 3 | 1.4 | Allele | 0.129 |
| NLRP3 | rs4353135 | TT |  | 49 | 31.4 | 36 | 34.0 | Codominant | **0.025** |
|  |  | GG |  | 24 | 15.4 | 29 | 27.4 | Dominant | 0.665 |
|  |  | GT |  | 83 | 53.2 | 41 | 38.7 | Recessive | **0.018** |
|  |  |  | G | 131 | 42.0 | 99 | 46.7 | Allele | 0.286 |
|  |  |  | T | 181 | 58.0 | 113 | 53.3 |  |  |
|  | rs35829419 | CC |  | 156 | 100.0 | 106 | 100.0 | Codominant | _ |
|  |  |  | C | 312 | 100.0 | 212 | 100.0 | Dominant | _ |
|  |  |  |  |  |  |  |  | Recessive | _ |
|  |  |  |  |  |  |  |  | Allele | _ |
|  | rs10754558 | CC |  | 49 | 31.4 | 37 | 34.9 | Codominant | 0.170 |
|  |  | GG |  | 24 | 15.4 | 24 | 22.6 | Dominant | 0.554 |
|  |  | CG |  | 83 | 53.2 | 45 | 42.5 | Recessive | 0.136 |
|  |  |  | C | 181 | 58.0 | 119 | 56.1 | Allele | 0.669 |
|  |  |  | G | 131 | 42.0 | 93 | 43.9 |  |  |
| CARD8 | rs2043211 | AA |  | 41 | 26.3 | 28 | 26.4 | Codominant | 0.202 |
|  |  | TT |  | 37 | 23.7 | 35 | 33.0 | Dominant | 0.098 |
|  |  | AT |  | 78 | 50.0 | 43 | 40.6 | Recessive | 0.981 |
|  |  |  | A | 160 | 51.3 | 99 | 46.7 | Allele | 0.303 |
|  |  |  | T | 152 | 48.7 | 113 | 53.3 |  |  |
| PTPN22 | rs33996649 | CC |  | 156 | 100.0 | 106 | 100.0 | Codominant | _ |
|  |  |  | C | 312 | 100.0 | 212 | 100.0 | Dominant | _ |
|  |  |  |  |  |  |  |  | Recessive | _ |
|  |  |  |  |  |  |  |  | Allele | _ |
|  | rs1310182 | GG |  | 3 | 1.9 | 1 | 0.9 | Codominant | 0.389 |
|  |  | AA |  | 123 | 78.8 | 77 | 72.6 | Dominant | 0.246 |
|  |  | AG |  | 30 | 19.2 | 28 | 26.4 | Recessive | 0.903 |
|  |  |  | G | 36 | 11.5 | 30 | 14.2 | Allele | 0.376 |
|  |  |  | A | 276 | 88.5 | 182 | 85.8 |  |  |
| SH2B3 | rs3184504 | CC |  | 156 | 100.0 | 104 | 98.1 | Codominant | 0.163 |
|  |  | CT |  | 0 | 0.0 | 2 | 1.9 | Dominant | 0.163 |
|  |  |  | C | 312 | 100.0 | 210 | 99.1 | Recessive | _ |
|  |  |  | T | 0 | 0.0 | 2 | 0.9 | Allele | 0.163 |
| STAT4 | rs10181656 | CC |  | 68 | 43.6 | 37 | 34.9 | Codominant | 0.070 |
|  |  | GG |  | 20 | 12.8 | 8 | 7.5 | Dominant | 0.159 |
|  |  | CG |  | 68 | 43.6 | 61 | 57.5 | Recessive | 0.175 |
|  |  |  | C | 204 | 65.4 | 135 | 63.7 | Allele | 0.688 |
|  |  |  | G | 108 | 34.6 | 77 | 36.3 |  |  |
|  | rs7574869 | TT |  | 20 | 12.8 | 8 | 7.5 | Codominant | 0.056 |
|  |  | GG |  | 69 | 44.2 | 37 | 34.9 | Dominant | 0.131 |
|  |  | GT |  | 67 | 42.9 | 61 | 57.5 | Recessive | 0.175 |
|  |  |  | G | 205 | 65.7 | 135 | 63.7 | Allele | 0.633 |
|  |  |  | T | 107 | 34.3 | 77 | 36.3 |  |  |
| TNFAIP3 | rs10499194 | CC |  | 128 | 82.1 | 104 | 98.1 | Codominant | **0.000** |
|  |  | TT |  | 2 | 1.3 | 0 | 0.0 | Dominant | **0.000** |
|  |  | CT |  | 26 | 16.7 | 2 | 1.9 | Recessive | 0.516 |
|  |  |  | C | 282 | 90.4 | 210 | 99.1 | Allele | **0.000** |
|  |  |  | T | 30 | 9.6 | 2 | 0.9 |  |  |
|  | rs2230926 | TT |  | 136 | 87.2 | 95 | 89.6 | Codominant | 0.548 |
|  |  | GT |  | 20 | 12.8 | 11 | 10.4 | Dominant | 0.548 |
|  |  |  | G | 20 | 6.4 | 11 | 5.2 | Recessive | _ |
|  |  |  | T | 292 | 93.6 | 201 | 94.8 | Allele | 0.561 |
|  | rs5029939 | CC |  | 134 | 85.9 | 93 | 87.7 | Codominant | 0.668 |
|  |  | CG |  | 22 | 14.1 | 13 | 12.3 | Dominant | 0.668 |
|  |  |  | C | 290 | 92.2 | 199 | 93.9 | Recessive | _ |
|  |  |  | G | 22 | 7.1 | 13 | 6.1 | Allele | 0.679 |
|  | rs6920220 | GG |  | 155 | 99.4 | 105 | 99.1 | Codominant | 1.000 |
|  |  | AG |  | 1 | 0.6 | 1 | 0.9 | Dominant | 1.000 |
|  |  |  | G | 311 | 99.7 | 211 | 99.5 | Recessive | _ |
|  |  |  | A | 1 | 0.3 | 1 | 0.5 | Allele | 1.000 |
| TRAF1 | rs10818488 | GG |  | 50 | 32.1 | 36 | 34.0 | Codominant | 0.948 |
|  |  | AA |  | 30 | 19.2 | 20 | 18.9 | Dominant | 0.746 |
|  |  | AG |  | 76 | 48.7 | 50 | 47.2 | Recessive | 0.942 |
|  |  |  | G | 176 | 56.4 | 122 | 57.5 | Allele | 0.796 |
|  |  |  | A | 136 | 43.6 | 90 | 42.5 |  |  |

SNP, single nucleotide polymorphism; Uncorrected p value calculated with chi-squared test; **Bold** highlights statistical significance (p < 0.05).
