# Supplementary material for: Inflammation-Related Gene Polymorphisms Associated With Primary Immune Thrombocytopenia
Source: Front Immunol. 2017 Jun 28;8:744. doi: 10.3389/fimmu.2017.00744 (PMC5487479; doi:10.3389/fimmu.2017.00744)
Supplement: Supplementary file 4 [file Table_4.DOC]

**Supplementary Table S4**. Association between inflammation-related SNPs and refractoriness of ITP.

| Gene | SNP | Genotype | Allele | Non-refractory ITP | | Refractory ITP | | Model / allele | Uncorrected p value |
| --- | --- | --- | --- | --- | --- | --- | --- | --- | --- |
|  |  |  |  | Count | % | Count | % |  |  |
| CD24 | rs52812045 | GG |  | 122 | 41.9 | 10 | 47.6 | Codominant | 0.878 |
|  |  | AA |  | 31 | 10.7 | 2 | 9.5 | Dominant | 0.610 |
|  |  | AG |  | 138 | 47.4 | 9 | 42.9 | Recessive | 1.000 |
|  |  |  | G | 382 | 65.6 | 29 | 69.0 | Allele | 0.652 |
|  |  |  | A | 200 | 34.4 | 13 | 31.0 |  |  |
| CD226 | rs763361 | CC |  | 137 | 47.1 | 12 | 57.1 | Codominant | 0.596 |
|  |  | TT |  | 28 | 9.6 | 1 | 4.8 | Dominant | 0.373 |
|  |  | CT |  | 126 | 43.3 | 8 | 38.1 | Recessive | 0.725 |
|  |  |  | C | 400 | 68.7 | 32 | 76.2 | Allele | 0.312 |
|  |  |  | T | 182 | 31.3 | 10 | 23.8 |  |  |
| FCRL3 | rs945635 | CC |  | 106 | 36.4 | 6 | 28.6 | Codominant | 0.650 |
|  |  | GG |  | 49 | 16.8 | 3 | 14.3 | Dominant | 0.469 |
|  |  | CG |  | 136 | 46.7 | 12 | 57.1 | Recessive | 1.000 |
|  |  |  | C | 348 | 59.8 | 24 | 57.1 | Allele | 0.735 |
|  |  |  | G | 234 | 40.2 | 18 | 42.9 |  |  |
|  | rs7528684 | GG |  | 49 | 16.8 | 3 | 14.3 | Codominant | 0.650 |
|  |  | AA |  | 106 | 36.4 | 6 | 28.6 | Dominant | 0.469 |
|  |  | AG |  | 136 | 46.7 | 12 | 57.1 | Recessive | 1.000 |
|  |  |  | G | 234 | 40.2 | 18 | 42.9 | Allele | 0.735 |
|  |  |  | A | 348 | 59.8 | 24 | 57.1 |  |  |
|  | rs3761959 | CC |  | 106 | 36.4 | 6 | 28.6 | Codominant | 0.667 |
|  |  | TT |  | 48 | 16.5 | 3 | 14.3 | Dominant | 0.469 |
|  |  | CT |  | 137 | 47.1 | 12 | 57.1 | Recessive | 1.000 |
|  |  |  | C | 349 | 60.0 | 24 | 57.1 | Allele | 0.719 |
|  |  |  | T | 233 | 40.0 | 18 | 42.9 |  |  |
|  | rs11264799 | CC |  | 190 | 65.3 | 15 | 71.4 | Codominant | 0.625 |
|  |  | TT |  | 11 | 3.8 | 0 | 0.0 | Dominant | 0.567 |
|  |  | CT |  | 90 | 30.9 | 6 | 28.6 | Recessive | 1.000 |
|  |  |  | C | 470 | 80.8 | 36 | 85.7 | Allele | 0.428 |
|  |  |  | T | 112 | 19.2 | 6 | 14.3 |  |  |
| IL2 | rs6822844 | GG |  | 291 | 100.0 | 21 | 100.0 | Codominant | _ |
|  |  |  | G | 582 | 100.0 | 42 | 100.0 | Dominant | _ |
|  |  |  |  |  |  |  |  | Recessive | _ |
|  |  |  |  |  |  |  |  | Allele | _ |
| IRF5 | rs2280714 | CC |  | 54 | 18.6 | 5 | 23.8 | Codominant | 0.767 |
|  |  | TT |  | 102 | 35.1 | 6 | 28.6 | Dominant | 0.547 |
|  |  | CT |  | 135 | 46.4 | 10 | 47.6 | Recessive | 0.760 |
|  |  |  | C | 243 | 41.8 | 20 | 47.6 | Allele | 0.457 |
|  |  |  | T | 339 | 58.2 | 22 | 52.4 |  |  |
|  | rs2004640 | GG |  | 164 | 56.4 | 10 | 47.6 | Codominant | 0.738 |
|  |  | TT |  | 23 | 7.9 | 2 | 9.5 | Dominant | 0.436 |
|  |  | GT |  | 104 | 35.7 | 9 | 42.9 | Recessive | 1.000 |
|  |  |  | G | 432 | 74.2 | 29 | 69.0 | Allele | 0.461 |
|  |  |  | T | 150 | 25.8 | 13 | 31.0 |  |  |
|  | rs10954213 | GG |  | 68 | 23.4 | 8 | 38.1 | Codominant | 0.316 |
|  |  | AA |  | 86 | 29.6 | 5 | 23.8 | Dominant | 0.576 |
|  |  | AG |  | 137 | 47.1 | 8 | 38.1 | Recessive | 0.129 |
|  |  |  | G | 273 | 46.9 | 24 | 57.1 | Allele | 0.200 |
|  |  |  | A | 309 | 53.1 | 18 | 42.9 |  |  |
| ITGAM | rs1143679 | GG |  | 287 | 98.6 | 21 | 100.0 | Codominant | 1.000 |
|  |  | AG |  | 4 | 1.4 | 0 | 0.0 | Dominant | 1.000 |
|  |  |  | G | 578 | 99.3 | 42 | 100.0 | Recessive | _ |
|  |  |  | A | 4 | 0.7 | 0 | 0.0 | Allele | 1.000 |
| NLRP3 | rs4353135 | GG |  | 66 | 22.7 | 5 | 23.8 | Codominant | 0.403 |
|  |  | TT |  | 88 | 30.2 | 9 | 42.9 | Dominant | 0.228 |
|  |  | GT |  | 137 | 47.1 | 7 | 33.3 | Recessive | 1.000 |
|  |  |  | G | 269 | 46.2 | 17 | 40.5 | Allele | 0.471 |
|  |  |  | T | 313 | 53.8 | 25 | 59.5 |  |  |
|  | rs35829419 | CC |  | 291 | 100.0 | 21 | 100.0 | Codominant | _ |
|  |  |  | C | 582 | 100.0 | 42 | 100.0 | Dominant | _ |
|  |  |  |  |  |  |  |  | Recessive | _ |
|  |  |  |  |  |  |  |  | Allele | _ |
|  | rs10754558 | CC |  | 91 | 31.3 | 7 | 33.3 | Codominant | 0.443 |
|  |  | GG |  | 54 | 18.6 | 6 | 28.6 | Dominant | 0.844 |
|  |  | CG |  | 146 | 50.2 | 8 | 38.1 | Recessive | 0.402 |
|  |  |  | C | 328 | 56.4 | 22 | 52.4 | Allele | 0.616 |
|  |  |  | G | 254 | 43.6 | 20 | 47.6 |  |  |
| CARD8 | rs2043211 | AA |  | 69 | 23.7 | 8 | 38.1 | Codominant | 0.142 |
|  |  | TT |  | 76 | 26.1 | 7 | 33.3 | Dominant | 0.470 |
|  |  | AT |  | 146 | 50.2 | 6 | 28.6 | Recessive | 0.140 |
|  |  |  | A | 284 | 48.8 | 22 | 52.4 | Allele | 0.654 |
|  |  |  | T | 298 | 51.2 | 20 | 47.6 |  |  |
| PTPN22 | rs33996649 | CC |  | 291 | 100.0 | 21 | 100.0 | Codominant | _ |
|  |  |  | C | 582 | 100.0 | 42 | 100.0 | Dominant | _ |
|  |  |  |  |  |  |  |  | Recessive | _ |
|  |  |  |  |  |  |  |  | Allele | _ |
|  | rs1310182 | GG |  | 7 | 2.4 | 0 | 0.0 | Codominant | 0.871 |
|  |  | AA |  | 222 | 76.3 | 16 | 76.2 | Dominant | 1.000 |
|  |  | AG |  | 62 | 21.3 | 5 | 23.8 | Recessive | 1.000 |
|  |  |  | G | 76 | 13.1 | 5 | 11.9 | Allele | 0.830 |
|  |  |  | A | 506 | 86.9 | 37 | 88.1 |  |  |
| SH2B3 | rs3184504 | CC |  | 289 | 99.3 | 21 | 100.0 | Codominant | 1.000 |
|  |  | CT |  | 2 | 0.7 | 0 | 0.0 | Dominant | 1.000 |
|  |  |  | C | 580 | 99.7 | 42 | 100.0 | Recessive | _ |
|  |  |  | T | 2 | 0.3 | 0 | 0.0 | Allele | 1.000 |
| STAT4 | rs10181656 | CC |  | 119 | 40.9 | 6 | 28.6 | Codominant | 0.055 |
|  |  | GG |  | 36 | 12.4 | 0 | 0.0 | Dominant | 0.266 |
|  |  | CG |  | 136 | 46.7 | 15 | 71.4 | Recessive | 0.174 |
|  |  |  | C | 374 | 54.3 | 27 | 64.3 | Allele | 0.997 |
|  |  |  | G | 208 | 35.7 | 15 | 35.7 |  |  |
|  | rs7574869 | GG |  | 121 | 41.6 | 6 | 28.6 | Codominant | **0.048** |
|  |  | TT |  | 36 | 12.4 | 0 | 0.0 | Dominant | 0.241 |
|  |  | GT |  | 134 | 46.0 | 15 | 71.4 | Recessive | 0.174 |
|  |  |  | G | 376 | 64.6 | 27 | 64.3 | Allele | 0.967 |
|  |  |  | T | 206 | 35.4 | 15 | 35.7 |  |  |
| TNFAIP3 | rs10499194 | CC |  | 211 | 87.6 | 21 | 100.0 | Codominant | 0.274 |
|  |  | TT |  | 2 | 0.8 | 0 | 0.0 | Dominant | 0.174 |
|  |  | CT |  | 28 | 11.6 | 0 | 0.0 | Recessive | 1.000 |
|  |  |  | C | 450 | 93.4 | 42 | 100.0 | Allele | 0.165 |
|  |  |  | T | 32 | 6.6 | 0 | 0.0 |  |  |
|  | rs2230926 | TT |  | 258 | 88.7 | 19 | 90.5 | Codominant | 1.000 |
|  |  | GT |  | 33 | 11.3 | 2 | 9.5 | Dominant | 1.000 |
|  |  |  | G | 33 | 5.7 | 2 | 4.8 | Recessive | _ |
|  |  |  | T | 549 | 94.3 | 40 | 95.2 | Allele | 1.000 |
|  | rs5029939 | CC |  | 254 | 87.3 | 18 | 85.7 | Codominant | 1.000 |
|  |  | CG |  | 37 | 12.7 | 3 | 14.3 | Dominant | 1.000 |
|  |  |  | C | 545 | 93.6 | 39 | 92.9 | Recessive | _ |
|  |  |  | G | 37 | 6.4 | 3 | 7.1 | Allele | 1.000 |
|  | rs6920220 | GG |  | 288 | 99.0 | 21 | 100.0 | Codominant | 1.000 |
|  |  | AG |  | 3 | 1.0 | 0 | 0.0 | Dominant | 1.000 |
|  |  |  | G | 579 | 99.5 | 42 | 100.0 | Recessive | _ |
|  |  |  | A | 3 | 0.5 | 0 | 0.0 | Allele | 1.000 |
| TRAF1 | rs10818488 | GG |  | 95 | 32.6 | 6 | 28.6 | Codominant | 0.498 |
|  |  | AA |  | 66 | 22.7 | 3 | 14.3 | Dominant | 0.700 |
|  |  | AG |  | 130 | 44.7 | 12 | 57.2 | Recessive | 0.533 |
|  |  |  | G | 320 | 55.0 | 24 | 57.1 | Allele | 0.786 |
|  |  |  | A | 262 | 45.0 | 18 | 42.9 |  |  |

SNP, single nucleotide polymorphism; Uncorrected p value calculated with chi-square test; **Bold** highlights statistical significance (p < 0.05).
